# Acute Thrombotic Occlusion of the Popliteal Artery following Knee Dislocation: A Case Report of Management, Local Unit Practice, and a Review of the Literature

**DOI:** 10.1155/2017/5346457

**Published:** 2017-01-26

**Authors:** Anthony Dean Godfrey, Fadi Hindi, Callum Ettles, Mark Pemberton, Perbinder Grewal

**Affiliations:** Department of Vascular Surgery, Queen Alexandra Hospital, Portsmouth Hospitals NHS Trust, Portsmouth, UK

## Abstract

Arterial complications following traumatic knee injury are relatively rare but mandate timely recognition and treatment to avoid significant comorbidity and medicolegal ramifications. In this report we describe a case of acute thrombotic occlusion of the popliteal artery occurring after knee dislocation, successfully repaired by intimal fixation and a limited venous patch reconstruction. We present a review of local practice in screening vascular injuries following knee dislocation, aligned with a review of the literature and considerations for practice.

## 1. Introduction

Popliteal artery injury following blunt trauma to the lower extremity has been shown to range from 28% to 46% [1–3]. Without timely recognition and treatment, significant comorbidity, including limb loss and ensuing medicolegal consequences of missed diagnosis, has been reported [4]. The popliteal artery is uniquely vulnerable to blunt extremity trauma due to its unique fixation and anatomic relationships [5]. Popliteal artery distraction injuries, with intima disruption, flap formation, and thrombus in situ formation, are frequently associated with knee dislocation following blunt trauma [1, 6].

This paper describes a case of knee dislocation with popliteal artery vascular injury and its timely recognition, investigation, and focused open surgical management.

## 2. Case Report

**Figure 1:**
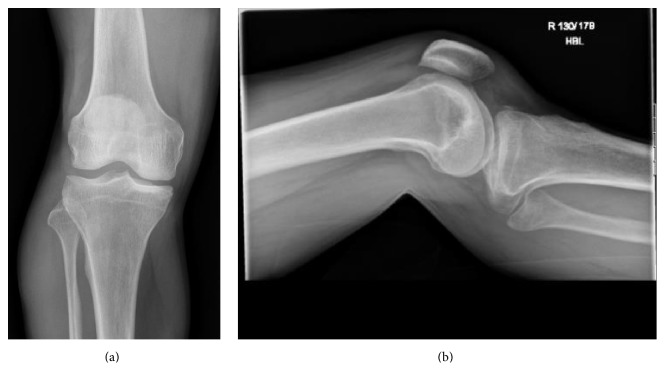
Plain radiology of right knee (25th of May 2016 @ 13:03).

A 58-year-old male mechanic was admitted via the emergency department (ED) one hour after bilateral knee injury with presumed spontaneous relocation of bilateral knee dislocations. Whilst standing at the front of one car working under the bonnet, a coworker started the vehicle (in gear) causing it to jolt forward pinning him against another car, causing a distraction crush injury to both knees (at the level of the vehicle bumpers). The patient was placed in bilateral vacuum splints and brought to hospital by ambulance. Plain radiography of the right knee (Figure 1) identified a small quantity of fluid in the suprapatellar recess with lateral joint space increase and medial tibial plateau irregularity, in keeping with a relocated lateral fracture dislocation of the patellofemoral joint with avulsion fracturing of the anteromedial tibial plateau.

**Figure 2:**
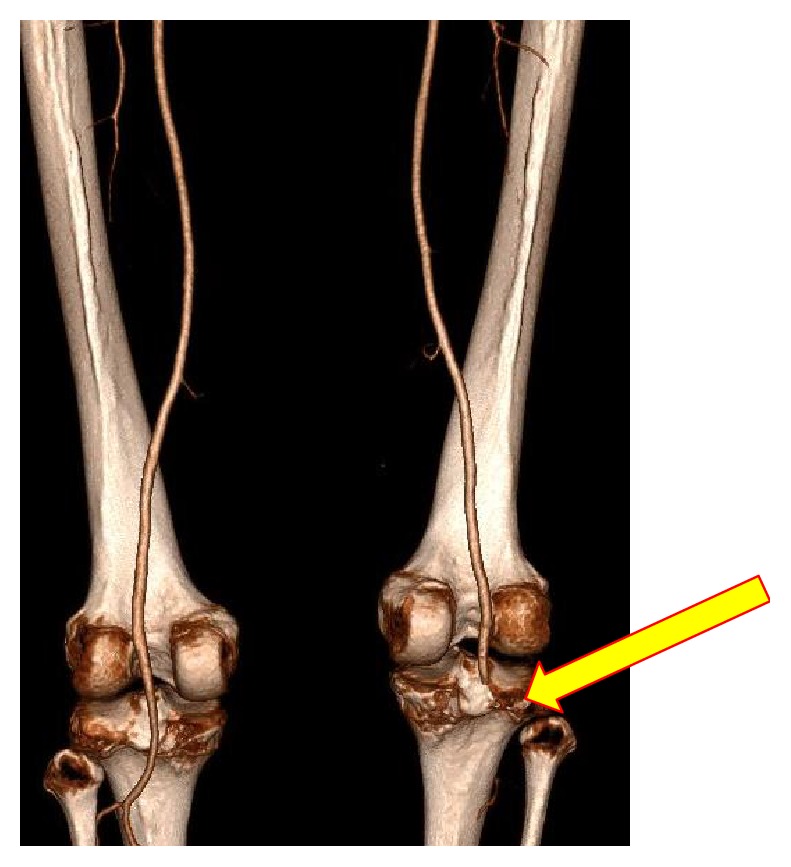
CT peripheral angiogram (25th of May 2016 @ 15:37), 3D reconstruction, posterior view displaying normal vasculature on left, and occlusion to right popliteal artery.

Despite significant distracting injuries, the ED clinicians quickly identified the absence of peripheral pulses on the right foot and alerted the vascular on call team. Clinical assessment identified normal pulses throughout the left lower limb in the presence of left ligamentous knee disruption and only a femoral pulse on the right with an asymmetric ABPI (0.9 versus 0.3, resp.), with an incomplete sensorimotor deficit below an unstable knee joint. Following administration of an intravenous heparin bolus (5000 international units), a CT angiogram [CTA] as shown in Figure 2 was performed to delineate the arterial vascular pathology and confirm deep venous patency in addition to bony injuries. The scan confirmed an acutely occluded right popliteal artery.

Following multidisciplinary case review (radiology, trauma and orthopaedics, ED, anaesthetics, and vascular surgery) and with patient informed consent, we proceeded to the emergency operating theatre for revascularisation of the right lower limb (@18:30).

Following our standard preprocedural case plan outline and World Health Organisation (WHO) safety surgery checklist, the great saphenous vein (GSV) position and patency were confirmed and marked under ultrasound for use as a vascular conduit. General anaesthesia was induced alongside prophylactic intravenous Co-amoxiclav (1.2 grams) followed by a urinary catheter. The patient was positioned supine, on our standard X-ray compatible surgical table, and the left leg was immobilised in a cricket pad splint. The right leg underwent hair removal over the incision site (over the GSV) and was prepared with 2% alcoholic chlorhexidine gluconate from the groin to ankle; the foot was placed in a Bogota bag for monitoring.

Using a standard medial approach to the popliteal vessels, an incision was placed 1-2 cm posterior to the medial border of the tibia, starting at the tibial tuberosity and extending distally. Subcutaneous fat and fascia were sharply divided, preserving GSV in the wound limits. To reach the popliteal fossa, gastrocnemius muscle was retracted dorsally and deep fascia divided* (occasionally pes anserinus and soleus may be divided for enhanced exposure)*. The popliteal artery was identified and controlled using silastic vessel slings at the below knee popliteal inflow component where a pulse was palpable and distally, at the anterior tibial artery origin, between which the popliteal vessel wall was thrombosed, creating a dark blue hue.

**Figure 3:**
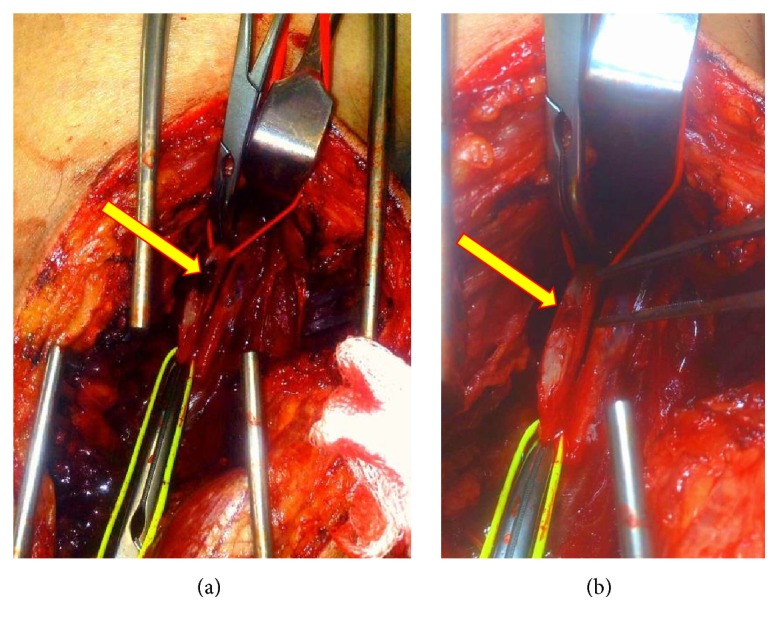
Intraoperative image of right popliteal artery demonstrating thrombus burden (a) and intimal distraction injury (b).

A longitudinal arteriotomy was performed over the area and extended into healthy vessel in both directions (2 cm each).  Figure 3 demonstrates the intraoperative finding of an intimal distraction injury over 1.5 cm length with a central thrombus burden. After confirming restoration of inflow and outflow, vessels were thoroughly flushed with heparinised saline (60 mL each direction) and controlled with small vascular clamps.

The intimal disruption was resected back to healthy vessel in both directions and tacked using multiple Prolene 7.0 sutures. The GSV was harvested in the wound edge over a 6 cm length and splayed for use as a tension-free patch on the popliteal artery using a Prolene 6.0 suture. Backflow and inflow were tested and flushed once again before completion of the patch anastomosis, with limb reperfusion following preparation and discussion with the anaesthetic team. Immediate arterial perfusion was noted with a corresponding blood pressure drop, end tidal carbon dioxide rise, and short run of tachyarrhythmia.

All four lower leg compartments were explored, through an extension of the medial tibial incision and the placement of a lateral incision anterior to the fibular. As all compartments remained soft after perfusion in the setting of a short ischaemic time, we elected to close incisions with interrupted nonabsorbable sutures (Ethilon 3.0) to expedite healing, whilst retaining the ability to release the sutures should compartment syndrome develop postoperatively. As a guide we advocate the following as indicators where fasciotomy is likely to be necessary:

- Severe ischaemia lasting longer than 4–6 hours
- Preoperative shock
- Firm compartments when palpated
- Decreased sensibility and/or motor function
- Compartment pressure > 30 mmHg
- Disproportionate pain localised distal to the vascular injury

Preoperatively, the decision was taken to avoid use of ligaclips and skin staples as the patient was under consideration for early ligamentous intervention and therefore required preoperative MRI. Use of such devices often causes* compatibility angst *in MR but also generates artefact which may negatively impact imaging interpretation for orthopaedic reconstruction.

Postoperatively, after revascularisation, the patient had a cricket pad splint applied with confirmation of joint space restoration with intraoperative fluoroscopy by our trauma and orthopaedic team. He was managed on continuous intravenous heparin (target APTR 2.0–4.0) for 3 days before switching to prophylactic low molecular weight heparin (Enoxaparin 40 mg once daily) and commenced on aspirin (75 mg once daily). He was medically fit for discharge by day 5 after operation with palpable pedal pulses but did not leave hospital until day 11 after operation due to equipment limitations for discharge.

**Figure 4:**
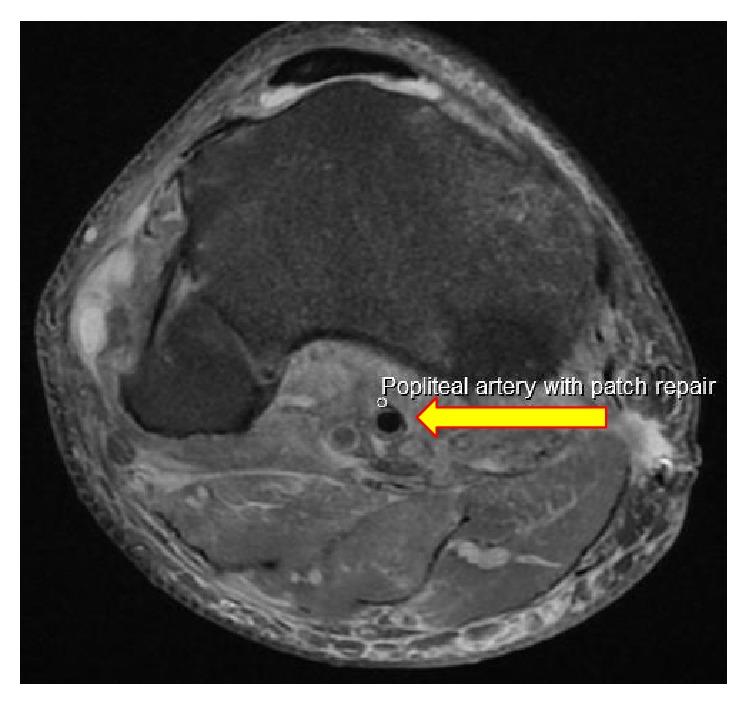
Postoperative MRI assessing multiligamentous disruption showing popliteal vessel patency and patch repair.

Subsequent MRI of the knees (Figure 4) identified on the left a complete ACL tear and medial collateral ligament. On the right, complete tear of PCL and LCL and posterolateral corner including popliteus and biceps femoris, partial tear of ACL, and a small impression fragment anteromedial corner of the medial tibial plateau were identified. Whilst offered an open direct reattachment of the ligamentous injuries at the acute phase, the patient declined in favour of pursuing a delayed reconstruction using autograph/allograft. He remains in bilateral IROM (integrated range of motion) braces.

## 3. Discussion

Blunt trauma to the lower extremity can cause popliteal artery injury in up to 46% of cases [1–3]. In the setting of trauma, popliteal artery reconstruction carries a 30% amputation rate, with concomitant venous injury in 60% of cases, fracture in 50%, and nerve injury in 50% also [7]. Diagnosis of an acute popliteal artery interruption is often obvious, although injuries may be missed if not actively investigated, particularly in the setting of spontaneously reduced knee dislocation.

Recommendations for the management of suspected vascular injuries in the lower limb have evolved from mandatory exploration of all suspected injuries to routine imaging [8]. In our unit, we advocate the use of duplex ultrasonography for screening of vascular pathologies where available, both for elective and emergency vascular workloads. Where duplex is unavailable outside of office hours or the pathology more extensive (cross speciality interest), we utilise computed tomography [CT] above magnetic resonance [MR], with very few (if ever) proceeding directly to diagnostic catheter arteriography (in isolation) for vascular malperfusion.

The use of cross-sectional imaging angiography is more often indicated after blunt trauma than penetrating because clinical examination is much more challenging in the setting of extensive soft tissue and nerve damage [8]. Thus CTA (most widely available) can aid localisation of the injured vessel and a targeted repair, such as, in this case, minimising unnecessary comorbidity associated with prolonged anaesthetic duration and operative risks of interposition or bypass grafting.

In a review of our contemporaneous practice (2015-current), we analysed a prospectively collated orthopaedic database for trauma admissions, cross referencing data with electronic imaging picture archiving and communication system (Sectra-PACS) and patient records. We identified seven knee dislocations (not including this case report); five were imaged for vascular pathology and only one was pathological, demonstrating popliteal occlusion requiring an interposition graft and external fixation in a patient with an incomplete sensorimotor deficit.

Clearly, injury of the popliteal artery is detrimental to distal limb perfusion and associated with poor outcomes, particularly in those with prolonged ischaemic time. The timely recognition of extremity arterial injury should be considered in the context of the following:

- Hard signs [9], including pulse absence (particularly nonsymmetrical), bruit or thrill, active or pulsatile haemorrhage, the six Ps of acute limb ischaemia (pain, pulseless, pallor, perishing cold, paraesthesia, and paralysis), compartment syndrome, a pulsatile, and/or expanding haematoma
- Soft signs [9], such as injury proximity to vessels, major single nerve deficit, nonexpanding haematoma, diminished pulses, midlimb joint dislocation, hypotension, and/or moderate blood loss at scene

**Figure 5:**
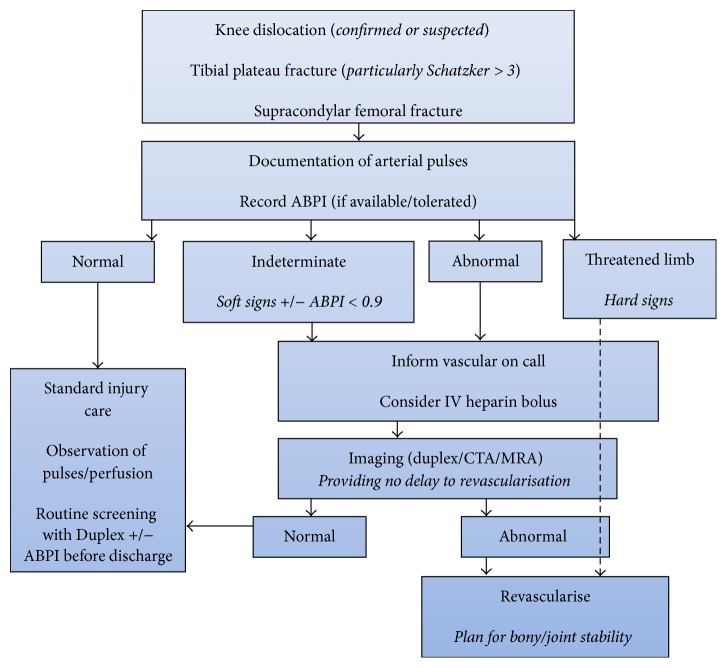
Suggested management algorithm of blunt force knee injuries.

A recent systematic review identified a lack of consensus among practitioners regarding the diagnostic and treatment algorithm for vascular injury in the context of knee dislocation [10]. We recommend that institutions utilise the management algorithm as shown in Figure 5 in the vascular investigation of blunt force injuries around the knee.

In patients with popliteal injury, the goal should be to permanently restore continuity of the artery without stenosis or tension. A previous study reported that simple thrombectomy alone was insufficient and 71% of patients required a revascularisation (bypass) procedure [11]. Therefore, the pathology type determines the choice of technique, varying from a few simple sutures to reconstruction with a patch, interposition, or bypass grafting. Autologous vein is the preferred graft material in such settings; it is infection resistant and flexible and allows elongation and vasodilatation in comparison to synthetic materials. Use of the ipsilateral GSV is favoured; however deep venous patency should be confirmed prior to its use. If injury is confirmed or suspected in the deep system, we recommend use of the contralateral great saphenous vein before considering harvest of arm vein or if not possible expanded polytetrafluoroethylene (ePTFE).

Early diagnosis and accurate initial therapy are essential for limb salvage in the context of popliteal injury following knee trauma which mandates an understanding of the underlying biomechanics of vessel injury in the popliteal, good working relationships across the multidisciplinary team and clear communication.
